# Preliminary study of the toxicity and radioprotective effects of zymosan in vitro and in vivo

**DOI:** 10.1186/s40360-021-00482-1

**Published:** 2021-03-17

**Authors:** Yue-zhi Zhang, Shu-jing Ge, Qing-zhen Leng, Jian-jun Ma, Han-chen Liu

**Affiliations:** 1 Laboratory of Biochemistry and Molecular Biology, Binzhou Medical University, Yantai, 264000 China; 2 Nursing Department, 970 Hospital of Chinese People’s Liberation Army, Yantai, 264002 China; 3 Cancer Non-Invasive Diagnosis and Treatment Center, 970 Hospital of Chinese People’s Liberation Army, No. 7, ZhiChu South Road, Yantai, 264002 China

**Keywords:** Zymosan, Radioprotection, Cytotoxicity, Apoptosis, Survival rate

## Abstract

**Background:**

This study aimed to confirm the cytotoxicity of zymosan in vitro and in vivo and determine the appropriate treatment time and the dose of zymosan.

**Methods:**

AHH-1 cells and HIECs were administered by 0, 20, 40, 80 or 160 μg/mL zymosan. The CCK-8 assay and flow cytometry were used to evaluate the cell viability and apoptosis 24 h, 48 h, and 72 h after administration. Furthermore, 12 h before irradiation, the cells were treated with 0, 5, 10, or 20 μg/mL zymosan and then irradiated with 4 Gy X-rays. Cell viability and apoptosis were measured by the CCK-8 assay and flow cytometry at 24 h. In addition, the protective effect of zymosan against radiation in vitro was compared to that of 20 μg/mL LPS*.* In vivo, weight, the spleen index, and the thymus index were measured to evaluate the toxicity of 0, 5, 10, 20, and 10 mg/kg zymosan. In addition, rats were treated with 0, 2, 4, 8, or 10 mg/kg zymosan and then irradiated with 7 Gy X-rays. The survival rate, organ index were evaluated. The protective effect of zymosan against radiation in vivo was compared to that of 10 mg/kg LPS a positive control*.*

**Results:**

The viability and apoptosis of cells treated with different doses and treatment times of zymosan were not different from those of control cells (*p* < 0.05). Furthermore, cell viability and apoptosis were clearly improved after zymosan preadministration (p < 0.05). The radioprotective effect of zymosan was dose-dependent. In addition, the viability of cells pretreated with zymosan was higher than that of cells pretreated with LPS, and the apoptosis rate of zymosan-treated cells was lower than that of cells pretreated with LPS (*p* < 0.05). In vivo*,* weight, the spleen index and the thymus index were significantly decreased by zymosan at a concentration of 20 mg/kg (*p* < 0.05). Further experiments showed that the concentration at which zymosan exerted radioprotective effects was 10 mg/kg. The survival curves in the irradiated rats were barely separated between the LPS treatment and zymosan treatment.

**Conclusion:**

Zymosan administration before radiation exposure significantly increased cell viability and the survival rates of rats.

**Supplementary Information:**

The online version contains supplementary material available at 10.1186/s40360-021-00482-1.

## Background

Humans are inevitably exposed to some radiation derived from various trace radionuclides, such as cosmic rays [1], buildings [2], and Wi-Fi radiation [3], but these natural sources of radiation rarely cause fatal radiation damage to human beings. However, the development and utilization of artificial radiation sources such as nuclear power stations, nuclear reactors, and nuclear weapons have forced mankind to face new life-threatening risks [4]. Safety issues with nuclear sources can result in the generation of ionizing radiation, which may cause fatal radiation damage to humans or other organisms [5]. In recent years, increasing attention has been paid to radiation-related research worldwide by patients, physicians and staff in radiation related departments [6]. At present, the best antiradiation drug approved by the FDA is WR-2721, which is used by the US Army [7]. However, due to its obvious side effects of nausea and vomiting, the use of WR-2721 is restricted to a certain extent [8]. At present, most radioprotective drugs and drugs used to treat radiation exposure in the research stage have some shortcomings such as unclear effects, unclear mechanisms or high toxicity, so they are limited to preventive administration and injection after radiation exposure has no obvious therapeutic effect.

Zymosan, a water-soluble polysaccharide, is typically prepared from the fungal wall of yeast (*Saccharomyces cerevisiae*). It contains a type of dextran linked by β-1,3 glycoside bonds, and can bind to toll-like receptor (TLR)2 on inflammatory cells [9, 10]. The use of TLR-2 agonist, like zymosan, has the potential to provide protection against radiation-induced bone marrow cell apoptosis [11]. Liu and colleagues have found bone marrow cells benefit from the activation of TLR4 and its in vivo ligands in radiation biology [12]. However, the cytotoxicity of zymosan is unclear, and appropriate treatment time and the dose of zymosan are needed to further analyze.

In this study, we analyzed the cytotoxicity of zymosan in vitro and in vivo and determined the appropriate treatment time and the dose of zymosan. The findings provide an experimental basis for the development of safe and effective radioprotective drugs in the future.

## Methods

### Cell culture

Human peripheral blood B lymphocytes (AHH-1 cells, BNCC331188) were purchased from BNBIO.com (Beijing, China), and human intestinal epithelial cells (HIECs, MZ-0792) were purchased from Mingzhoubio.com (Zhejiang, China). The cells were cultured in DMEM containing 10% fetal bovine serum at 37 °C and 5% CO_2_.

### Cell counting kit (CCK)-8 assay

Cells (10^3^ cells/well) were cultured in an incubator containing 5% CO_2_ at 37 °C for 24 h, and then 10 μL of CCK-8 solution (GipBio, Shanghai, China) was added and mixed well. Cells were then incubated for 4 h with oscillation, followed by reading with microplate reader (Synergy H1, BioTek, USA). The absorbance of each well at 450 nm was measured by normalization to the blank control. The cell survival rate was calculated based on the following formula: cell survival rate (%) = [(As-Ab)/(Ac-Ab)] × 100.

As = Absorbance of treatment wells.

Ab = Absorbance of blank.

Ac = Absorbance of Control wells.

### Flow cytometry

The Annexin V-FITC/PI Kit (CA1020, Solarbio, Beijing, China) was used to detect cell apoptosis. Cells cultured for 24 h (1 × 10^6^ cells) were collected and washed with precooled phosphate-buffered saline (PBS, pH 7.2–7.4, Mlbio, Shanghai, China). The cells were suspended in 1 mL of 1× binding buffer containing Ca^2+^ and centrifuged at 300×g for 10 mins. Then, the cell concentration was adjusted to 1 × 10^6^ cells/mL with 1 mL of 1× binding buffer. A total of 100 μL of the cell solution was added to 5 μL of annexin V-FITC and incubated in the dark for 10 min at room temperature. Then, 5 μL of propidium iodide (PI) was added, and the cells were incubated for 5 min. Finally, the volume of the cell suspension was adjusted to 500 μL with PBS and evaluated by flow cytometry (1040, ACEA NovoCyteTM, USA) within one hour. The results were evaluated by Cell Quest software (Version 5.1, BD Biosciences).

### Effect of zymosan on cell toxicity

AHH-1 and HIEC cells were administered 0, 20, 40, 80 or 160 μg/ml zymosan (tlrl-zyn, InvoGen). The CCK-8 assay and cell flow cytometry were used to evaluate cell viability and apoptosis 24 h, 48 h, and 72 h after administration to determine the dose-limiting toxicity of zymosan.

### Determination of the dose at which zymosan exerts radioprotective effects on cell

Twelve hours before irradiation, cells were treated with 0, 5, 10, or 20 μg/ml zymosan and then irradiated with 4 Gy X-rays at a dose rate of 0.25 Gy/min (Synergy, Elekta, Beijing, China). The CCK-8 assay and flow cytometry were utilized to evaluate cell viability and apoptosis at 24 h to determine the optimal dose of zymosan.

### Comparison of the radioprotective effects of lipopolysaccharide and zymosan in vitro

Cells were randomly divided into 4 groups: the normal control (control) group (cells were normally cultured), irradiation only (model) group (cells were irradiated with 4 Gy radiation), LPS group (cells were treated with 20 μg/ml LPS 12 h before being irradiated), and zymosan group (cells were treated with 20 μg/ml zymosan 12 h before being irradiated).

### Animals

A total of 120 male Sprague Dawley rats weighing 180 ± 20 g (aged 6–8 weeks) were purchased from Jinan Pengyue Experimental Animal Co., Ltd. (scxk (Lu) 20,190,003). The rats were housed at normal temperature (22 ± 2 °C) and humidity (55 ± 5%). Standard diet for laboratory (Jinan Pengyue, Shandong, China) and water were freely provided, and the animals were housed under a 12-h light/dark cycle. The rats were adaptively fed for 1 week. The animal experiments were conducted following the guidelines of the National Institutes of Health (NIH pub. No. 85–23, revised 1996) and were approved by the Animal Protection and Use Committee of Binzhou Medical University.

### Toxic effects of zymosan on rats

Zymosan (0, 5, 10, 20, or 40 mg/kg) was sterilely injected intraperitoneally, which was dissolved in sterile normal saline. Then, the rats were fed routinely and observed for 21 days. Weight changes were observed weekly. The relative weight changes were calculated by (Treatment group-Control)/(Control group) * 100. After 21 days, the rats were euthanized with 3% pentobarbital sodium (150 mg/kg). The spleen, thymus and liver were removed and immediately weighed, and the organ index was calculated by the following formula: Organ index = organ weight/body weight [13].

### Determination of the appropriate dose of zymosan for radioprotection of rats

Twenty-four hours before irradiation, the rats were intraperitoneally injected with 0, 2, 4, 8 or 10 mg/kg zymosan and then irradiated with 7 Gy X-ray at a dose rate of 1.0 Gy/min. Then, they were fed routinely and observed for 21 days. The survival of the rats was assessed each day. If animals were moribund, they were humanely euthanized. After 21 days, the rats were euthanized with 3% pentobarbital sodium (150 mg/kg). The spleen index and thymus index were calculated.

### Comparison of the radioprotective effects of lipopolysaccharide and zymosan in vivo

Twenty-four rats were randomly divided into 4 groups, 6 in each group: the normal control (control) group (the rats were normally fed), irradiation only (model) group (the rats were irradiated with 7 Gy X-ray at a dose rate of 1.0 Gy/min), LPS group (the rats were intraperitoneally injected with 10 mg/kg LPS 24 h before being irradiated), and zymosan group (the rats were intraperitoneally injected with 10 mg/kg zymosan 12 h before being irradiated). If animals were moribund, they were humanely euthanized. The survival of the rats was assessed each day. After 21 days, the rats were euthanized with 3% pentobarbital sodium (150 mg/kg). The spleen index and thymus index were calculated.

### Statistical analysis

Prism 5.01 statistical analysis software was used for data processing, and the results are expressed as the mean ± standard deviation (^−^X ± SD). Data from multiple groups were assessed by one-way analysis of variance (ANOVA), and Tukey’s test was used for subsequent analysis. *p* < 0.05 indicated a significant difference.

## Results

### Effect of zymosan on cell viability

**Fig. 1:**
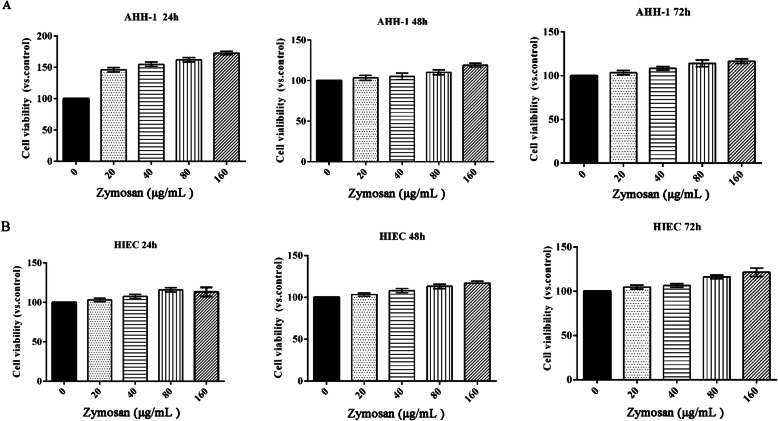
The effect of zymosan on cell viability. **a** The viability of AHH-1 cells treated with different concentrations of zymosan (0, 20, 40, 80, or 160 μg/mL) for 24, 48 or 72 h. **b** The viability of HIECs treated with different concentrations of zymosan (0, 20, 40, 80, or 160 μg/mL) for 24, 48 or 72 h. The test was repeated 3 times. The data are expressed as the^−^X ± SD. ANOVA was used to analyze the data from the different groups

To clarify the effect of zymosan on cell viability, AHH-1 cells and HIECs were treated by different concentrations of zymosan (0, 20, 40, 80 or 160 μg/mL). As demonstrated in Fig. 1a, the viability of AHH-1 cells was not affected upon treatment with different concentrations of zymosan. The viability of the AHH-1 cells was not affected at 24, 48 or 72 h. Similarly, the viability of the HIECs was not affected upon treatment with different concentrations of zymosan for different periods (Fig. 1b). These results showed that zymosan did not affect cell viability.

### Effect of zymosan on cell apoptosis

**Fig. 2:**
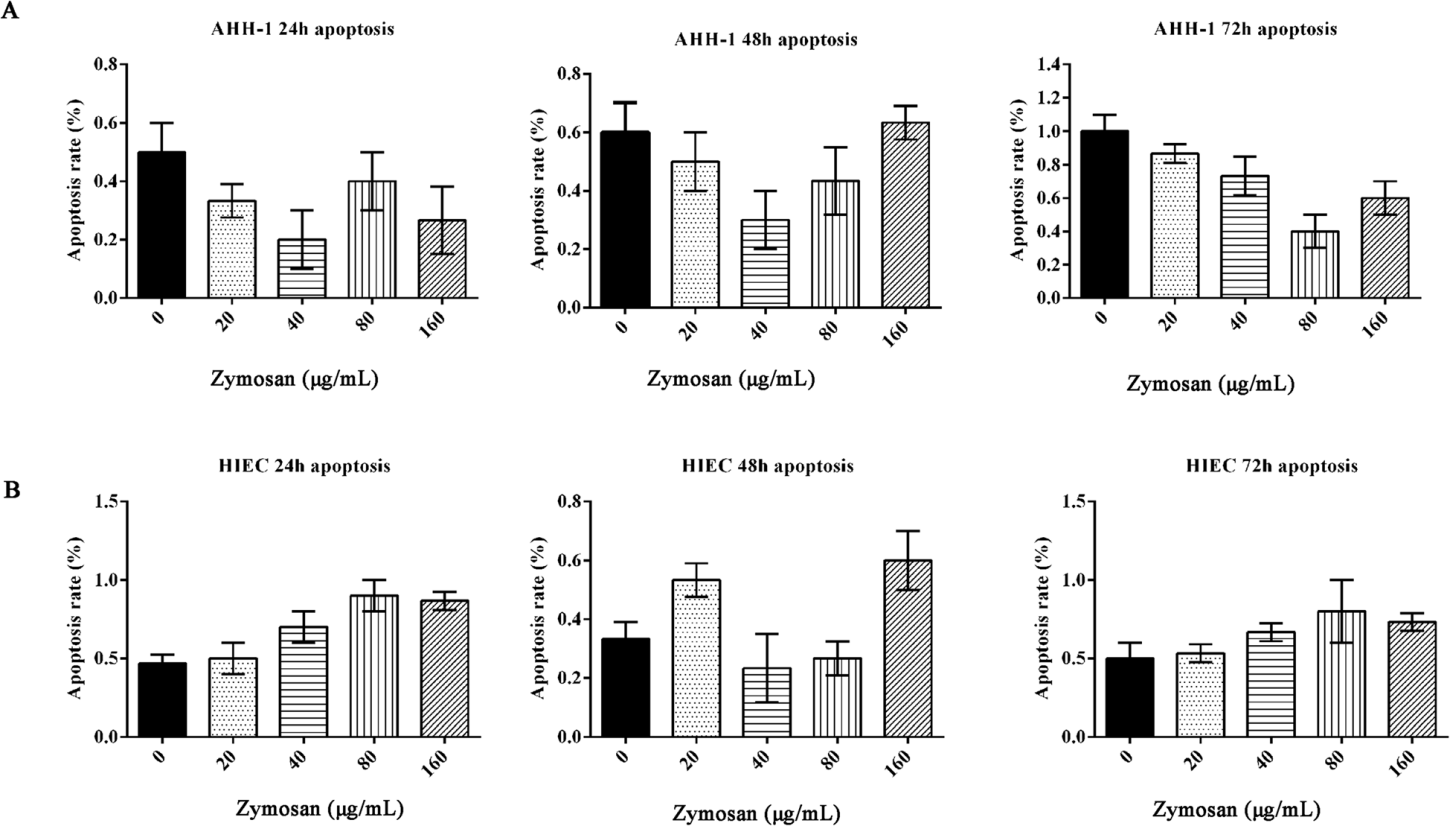
The effect of zymosan on cell apoptosis. **a** The apoptosis rate of AHH-1 cells treated with different concentrations of zymosan (0, 20, 40, 80, or 160 μg/mL) for 24 h, 48 h or 72 h. **b** The apoptosis rate of HIECs treated with different concentrations of zymosan (0, 20, 40, 80, or 160 μg/mL) for 24 h, 48 h or 72 h. The test was repeated 3 times. The data are expressed as the^−^X ± SD. ANOVA was used to analyze the data from the different groups

To further confirm that zymosan is not toxic to cells, the apoptosis of AHH-1 and HIEC cells was evaluated. Figure 2a shows the apoptosis rates of AHH-1 cells treated with 20, 40, 80 or 160 μg/mL zymosan for 24, 48, or 72 h. There were no clear differences among the different groups of cells (*p* > 0.05). The apoptosis rates of HIECs are shown in Fig. 2b. Similarly, the apoptosis rate of HIECs was not affected upon treatment with different concentrations of zymosan for different periods. The apoptosis results are shown in the supplementary file.

### The appropriate dose of zymosan for radioprotection of cells

**Fig. 3:**
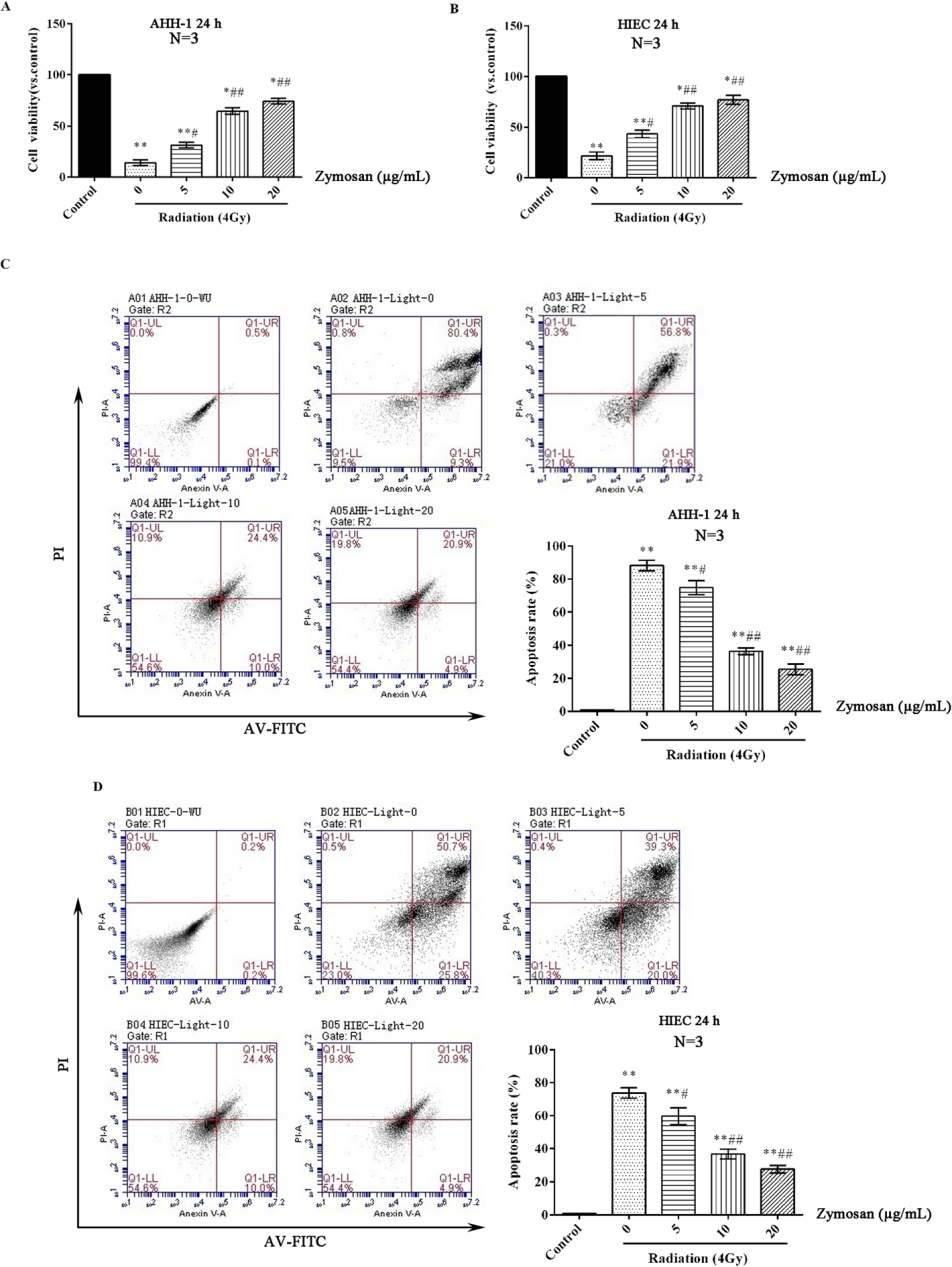
The radioprotective effects of zymosan on cells. Cells were treated with different concentrations of zymosan (0, 5, 10, or 20 μg/mL) 12 h before being treated with 4 Gy radiation. **a** The viability of AHH-1 cells after 24 h. **b** The viability of HIECs after 24 h. **c** The apoptosis rate of AHH-1 cells after 24 h. **d** The apoptosis rate of HIECs after 24 h. Compared with the control group, **p < 0.01; compared with the 0 μg/mL zymosan group, #*p* < 0.05, ##*p* < 0.01. The test was repeated 3 times. The data are expressed as the^−^X ± SD. ANOVA was used to analyze the data from the different groups

The radioprotective effects of zymosan pretreatment on AHH-1 cells and HIECs are shown in Fig. 3. The cells were administered 0, 5, 10 or 20 μg/mL zymosan 12 h before being irradiated. The viability of cells exposed to 4.0 Gy radiation was obviously decreased compared with that of control cells (*p* < 0.05, Fig. 3a, b), while the apoptosis rate of the irradiated cells was significantly increased (p < 0.05, Fig. 3c, d). The protective effect of zymosan on cells was dose-dependent. The higher the concentration of zymosan, the higher the viability of AHH-1 cells and HIECs (Fig. 3a, b) and the lower the apoptosis rate of AHH-1 cells and HIECs (Fig. 3c, d). Given the lower apoptosis rates, zymosan was used at a concentration of 20 μg/mL for further in vitro testing.

### Comparison of the radioprotective effects of LPS and zymosan on cells

**Fig. 4:**
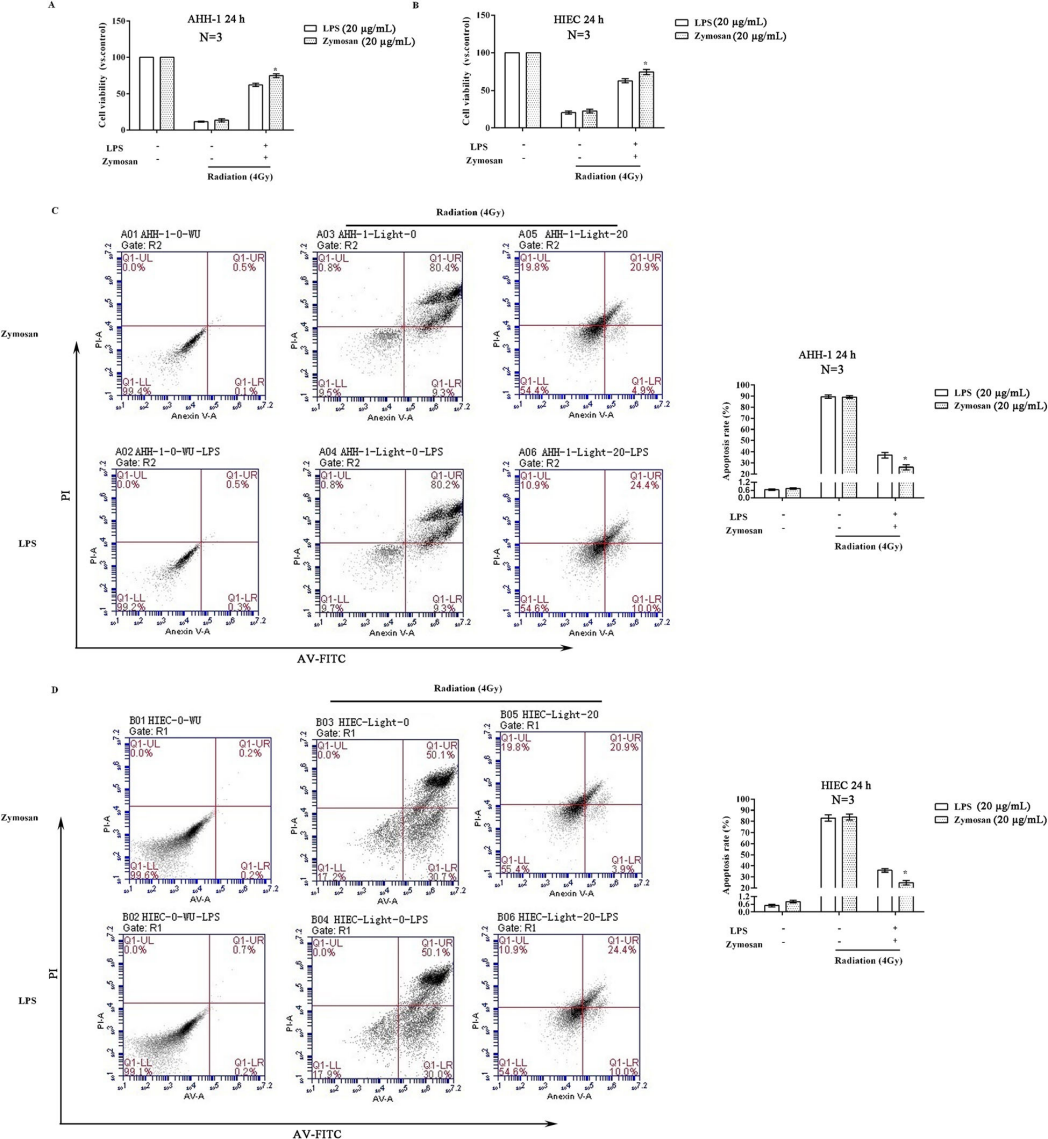
Comparison of the protective effects of LPS and zymosan against radiation in cells. Cells were treated with LPS (20 μg/mL) or zymosan (20 μg/mL) 12 h before being treated with 4 Gy radiation. **a** The viability of AHH-1 cells after 24 h. **b** The viability of HIECs after 24 h. **c** The apoptosis rate of AHH-1 cells after 24 h. **d** The apoptosis rate of HIECs after 24 h. Compared with the LPS group, *p < 0.05. The test was repeated 3 times. The data are expressed as the^−^X ± SD. ANOVA was used to analyze the data from the different groups

Cells treated with zymosan were demonstrated a lower cell death than those treated with LPS. As shown in Fig. 4a and b, the viability of AHH-1 cells and HIECs significantly increased after pretreatment with LPS or zymosan (20 μg/mL), and the cell viability of the zymosan pretreatment group was higher than that of the LPS pretreatment group (*p* < 0.05). The cell apoptosis assay (Fig. 4c, d) revealed that the apoptosis rates of AHH-1 cells and HIECs were significantly reduced after pretreatment with LPS and zymosan and that the apoptosis rate of cells pretreated with zymosan was lower than that of cells pretreated with LPS (*p* < 0.05). For radiation protection, this data showed that cells treated with zymosan showed a higher cell activity than those treated with LPS*.*

### Effect of zymosan on rat weight and organ indexes

**Fig. 5:**
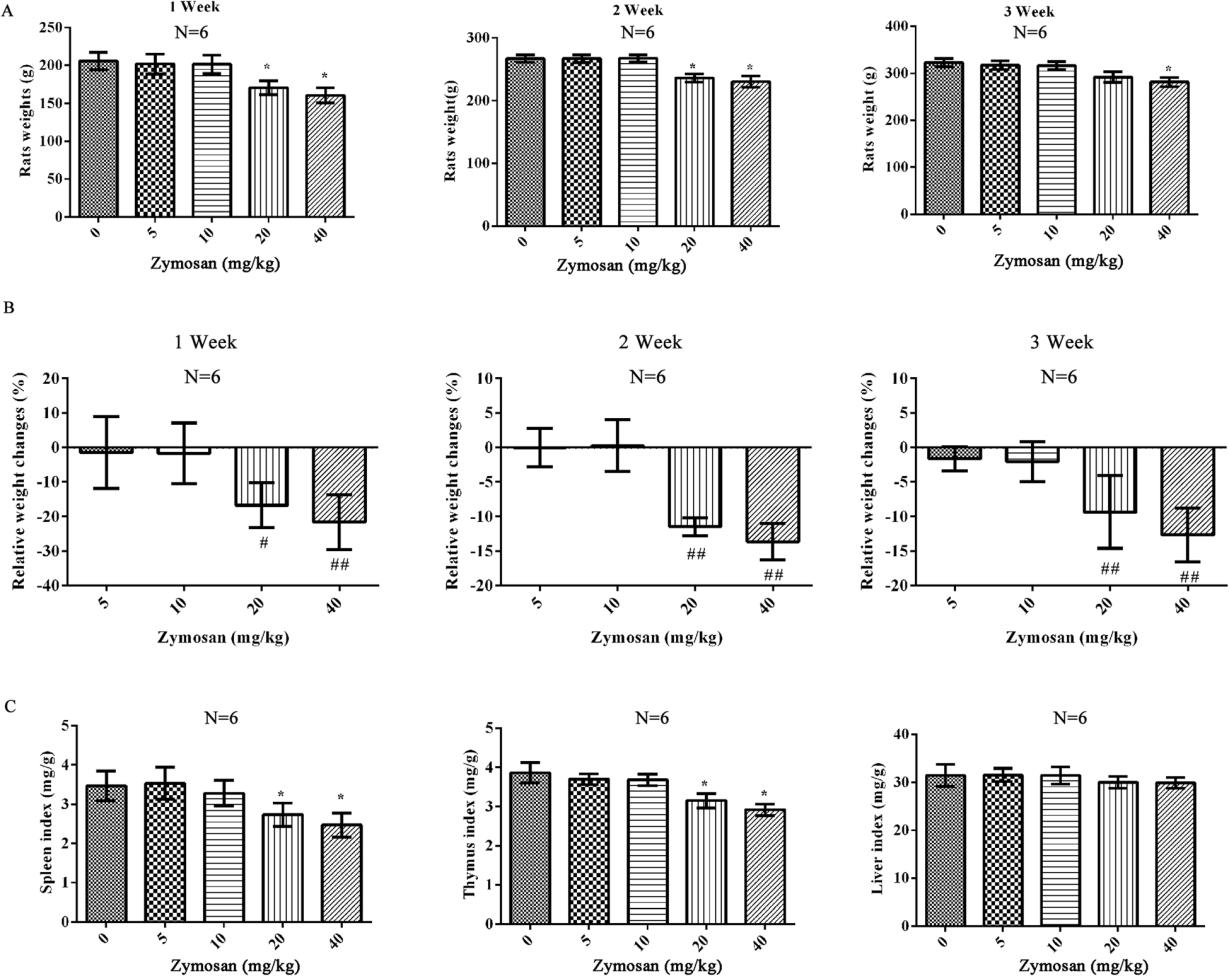
The effect of zymosan on rat weight, organs index. **a** The body weights of rats treated with different concentrations of zymosan (0, 5, 10, 20, or 40 mg/kg) for 1 week, 2 weeks or 3 weeks. **b** The relative weight changes were analyzed for1 week, 2 weeks or 3 weeks after treated with different concentrations of zymosan (0, 5, 10, 20, or 40 mg/kg). **c** The spleen index, thymus index and liver index after treatment with different concentrations of zymosan (0, 5, 10, 20, or 40 mg/kg). Compared with the 0 mg/kg zymosan group, *p < 0.05, **p < 0.01; Compared with 5 mg/kg zymosan group, #p < 0.05, ##p < 0.01. There were 6 rats in each group. The data are expressed as the^−^X ± SD. ANOVA was used to analyze the data from the different groups

In vivo, after different concentrations of zymosan (0, 5, 10, 20, 40 mg/kg) injection, the body weight, relative weight changes, spleen index, thymus index and liver index were observed for 3 weeks. The results showed that there was no significant difference in body weight between rats intraperitoneally injected with 5 or 10 mg/kg zymosan and control rats (*p* > 0.05, Fig. 5a). At a concentration of 20 mg/kg, zymosan significantly decreased the body weights of the rats (*p* < 0.05), and 40 mg/kg zymosan further decreased the body weights of the rats (Fig. 5a). Similarly, there was no difference in relative weight changes between 5 mg/kg and 10 mg/kg zymosan treatment (*p* > 0.05, Fig. 5b). Compared with 5 mg/kg zymosan, the relative weight changes were clearly decreased after 20 or 40 mg/kg zymosan injection (*p* < 0.05). The spleen index, thymus index and liver index were analyzed (Fig. 5c), and no significant difference in the spleen index or thymus index was found between rats intraperitoneally injected with 5 or 10 mg/kg zymosan and control rats (p > 0.05). At a concentration of 20 mg/kg, zymosan significantly decreased the spleen index and thymus index of the rats (*p* < 0.05), and 40 mg/kg zymosan further decreased the spleen index and thymus index when compared with control rats (p < 0.05, Fig. 5c).However, there was no significant difference in liver index among groups (p > 0.05).

### Dose at which zymosan exerts radioprotective effects in rats

**Fig. 6:**
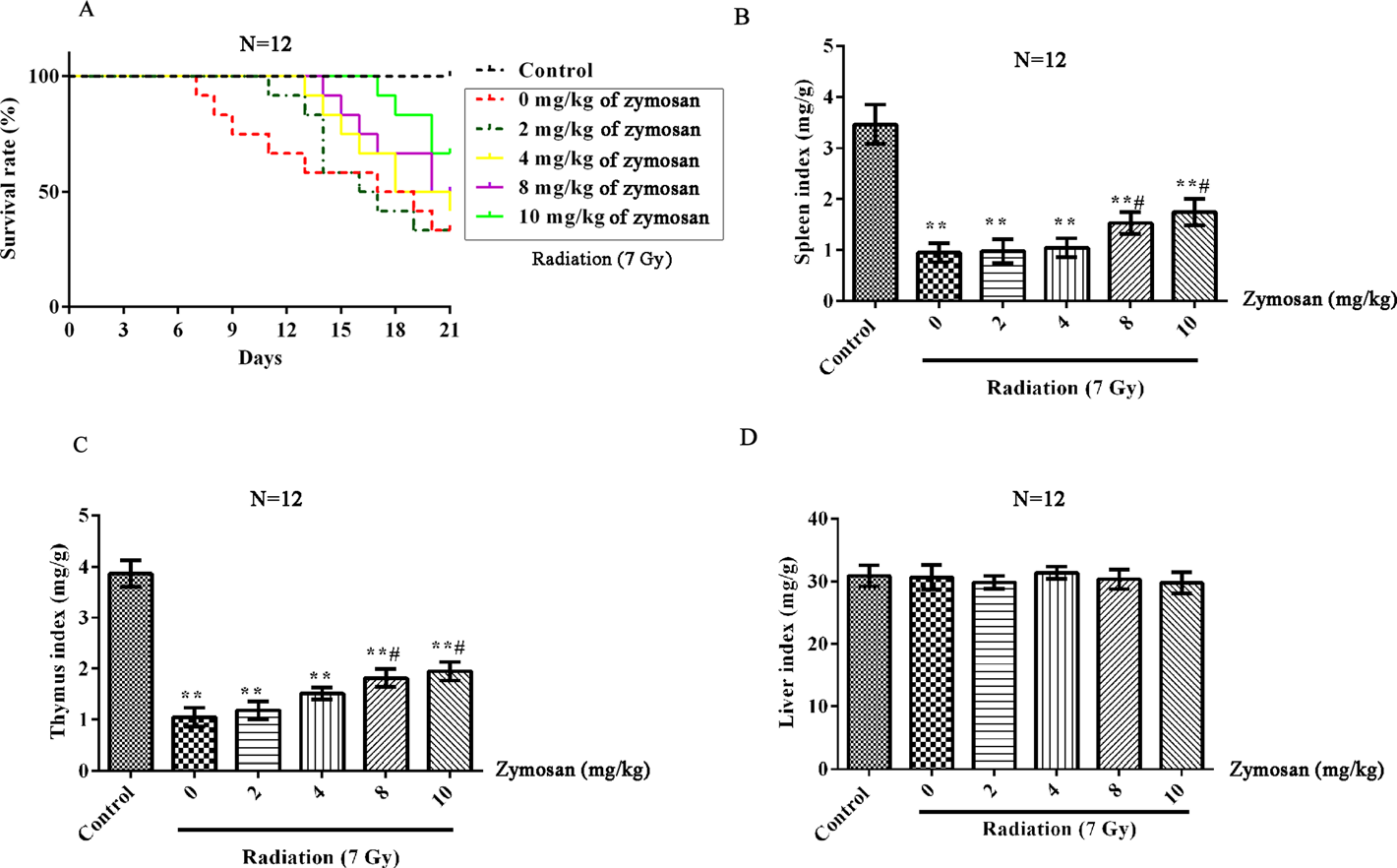
The radioprotective effects of zymosan in rats. Ats were treated with different concentrations of zymosan (0, 2, 4, 8, or 10 mg/kg) 24 h before being treated with 7.0 Gy irradiation. **a** The survival rate of rats after 21 d. **b** The spleen index; **c** The thymus index; **d** The liver index. Compared with the control group, **p < 0.01; Compared with the 0 mg/kg zymosan group, #p < 0.05. There were 12 rats in each group. The data are expressed as the^−^X ± SD. ANOVA was used to analyze the data from the different groups

The rats were administered 0, 2, 4, 8 or 10 mg/kg zymosan 24 h before being irradiated. The survival rate, spleen index and thymus index of rats exposed to 7.0 Gy radiation were obviously lower than those of control rats (*p* < 0.01, Fig. 6). The protective effect of zymosan on rats was dose-dependent. The higher the concentration of zymosan, the higher the survival rate, spleen index and thymus index of the rats. At a concentration of 8 mg/kg, zymosan significantly decreased the spleen index and thymus index of the rats (p < 0.05). Given higher survival rate, zymosan was used at a concentration of 10 mg/kg for further in vivo testing.

### Comparison of the radioprotective effects of LPS and zymosan in vivo

**Fig. 7:**
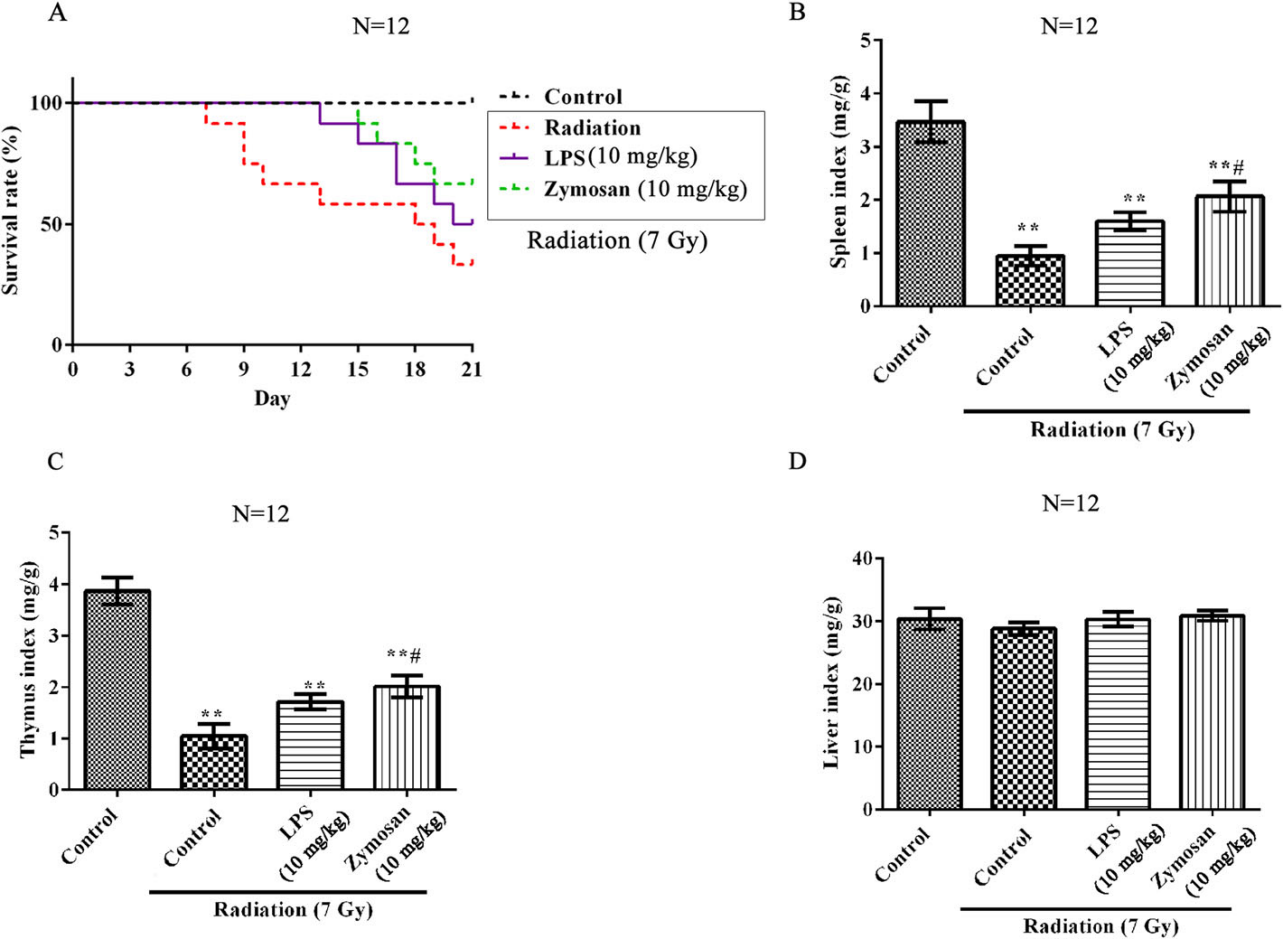
Comparison of the protective effects of LPS and zymosan against radiation in rats. Rats were treated with LPS (10 mg/kg) or zymosan (10 mg/kg) 24 h before being treated with 7.0 Gy irradiation. **a** The survival rate of rats after 21 d. **b** The spleen index; **c** The thymus index; **d** The liver index. Compared with the control group, **p < 0.01; Compared with the LPS-treated group, #p < 0.05. There were 12 rats in each group. The data are expressed as the^−^X ± SD. ANOVA was used to analyze the data from the different groups

As shown in Fig. 7, the survival rate, spleen index and thymus index were significantly decreased in zymosan- and LPS-treated rats compared with control rats (p < 0.01). LPS and zymosan (10 mg/kg) pretreatment prior to radiation exposure significantly increased the survival rate, spleen index and thymus index. Furthermore, there was no clear difference among groups in liver index (*p* > 0.05).

## Discussion

Nuclear radiation injuries mainly involve the destruction of genes and biological macromolecular structures and the degeneration or necrosis of tissue cells caused by ionizing radiation to cause tissue and organ dysfunction [14, 15]. The mechanisms for the occurrence and development of radiation injury have been analyzed thoroughly, but no ideal treatment has been identified. At present, there are two main strategies for protecting against ionizing radiation: one is external protection, that is, the damaging effects of rays on the body are reduced by using appropriate shielding materials, and the other way is internal protection, which means the appropriate antiradiation drugs are developed. Antiradiation drugs can counteract radioprotective effects before or after radiation exposure [16, 17]. Currently, most of the drugs used for the treatment of radiation-induced damage are western medicines, and they are mainly used for irradiation prevention and early treatment after irradiation, but some adverse reactions are usually accompanied when they are used, so they are not suitable for long-term use. However, repairing the body after radiation damage needs long-term treatment. Therefore, it is imperative to search for antiradiation drugs that can be orally administered with high efficiency, low toxicity, and few side effects and to explore the mechanisms underlying radioprotection.

Recently, some progress towards the research of antiradiation drugs has been made. The potency of sulfur-containing radiopreventive drugs (cysteamine S-phosphate sodium salt, N-acetylcysteine) is generally low [18, 19]. The potency of vitamins and hormones is equivalent to that of sulfur-containing drugs, but their effective doses are thousands of times higher than the physiological concentrations, and also the side effects associated with their long-term use are not easy to overcome [20, 21]. Zymosan is a natural polysaccharide β-glucan that is present in the yeast cell wall. It can exert strong effects and generally be free from side-effects. Zymosan has been shown to combine with receptors on the surface of immune cells to regulate immune responses and enhance the immune activity of cells [22]. Taghavi et al. also found that zymosan had the antitumor activity and played a positive role in inhibiting the progression of melanoma by regulating the expression of TLR-2, TLR-4 and tumor necrosis factor-α [23]. Although it has been reported that fungal beta glucan and mushroom β-glucan can improve the survival rate of irradiated mice [24, 25], there has been little research on the antiradiation effect of zymosan. In this research, based on cell and animal tests, the radioprotective effect of zymosan on cells and rats irradiated for different time periods was studied, and the appropriate dose of zymosan was preliminarily determined. We found that zymosan administration before radiation exposure significantly increased cell viability and the survival rates of rats.

Nevertheless, our results demonstrated that zymosan treatment decreased the weight of rats at a concentration of 20 mg/kg or 40 mg/kg. At the same time, the spleen index and thymus index were also clearly decreased at a concentration of 20 mg/kg or 40 mg/kg. The organ index didn’t capture the effects on the organ entirely. Specifically, changes in the cell structure or histopathology might help to explain the toxicity of zymosan in the organs. In further study, the pathological observation of organs is necessary to analyze the toxicity of zymosan.

The survival curves in the irradiated rats were barely separated between LPS treatment and zymosan treatment. Furthermore, LPS acted as protection in the cell viability after radiation. Interestingly, the values of cell viability and organs indexes were higher in the zymosan treatment than LPS treatment after radiation. Although zymosan has demonstrated potential as a prophylactic radiation treatment in this study, it still needs further researches to mitigate the noted toxicity. The combination of more agents may increase the radiation protection effect, but this view is only our speculation. The above results have shown that zymosan has an effective protection against ionizing radiation, which is simply as a preventative therapy. However, more researches are needed to explain why zymosan decreased spleen/thymus indices to produce a protective effect in the radiation treatment. The difference in radiosensitivity between genders has been well documented [26]. Klein et al. found women with comparatively lower radiation doses were more successful in fenestrated endovascular aneurysm repair [27]. Because of gender diversity on radiation dose, only male rats were chosen in this study, which is also a limitation of the study. In addition, Missig and colleagues found that prenatal TLR7 activation could induce a maternal inflammatory response and fewer female offspring, that were substantially different—and sometimes opposite to—those seen when targeting TLR3 and/or TLR4 [28]. This reveals that male and female animals as some key differences have been seen in the sensitivity to TLR activation.

However, the magnitude and severity of nuclear radiation’s harm to human health also depend on factors such as the type of radiation, radiation dose rate, dose absorbed by the body and individual sensitivity. Different types of nuclear radiation have significant differences in relative biological effects on organisms. Therefore, in the future, we will address the above issues and provide basic biological data for the development of effective radioprotective drugs.

## Conclusions

In summary, zymosan pretreatment significantly increased cell viability and the survival rate of rats, but the high dose of zymosan might be toxic to rats. In the further, the pathological observation of organs is necessary to analyze the toxicity of zymosan. There are still many uncertainties regarding the utilization of zymosan for the treatment of radiation-induced toxicity, such as how to improve the efficacy of zymosan as a preventative therapy and what is its function on those who are exposed to radiation.

## Supplementary Information

**Additional file 1.**

## Data Availability

The datasets used and analyzed during the current study are available from the corresponding author on reasonable request.

## Abbreviations

TLR: Toll-like receptor
PRRs: Pattern recognition receptors
NF-κB: Nuclear factor kappa beta
MyD88: Myeloid differentiation factor 88
IL: Interleukin
TNF: Tumor necrosis factor
LPS: Lipopolysaccharide
