# Supplementary material for: Preliminary study of the toxicity and radioprotective effects of zymosan in vitro and in vivo
Source: BMC Pharmacol Toxicol. 2021 Mar 17;22:16. doi: 10.1186/s40360-021-00482-1 (PMC7968253; doi:10.1186/s40360-021-00482-1)

1. AHH-1 and HIEC cells were respectively administered to zymosan at 0, 20, 40, 80 and 160 μg/ml at 24h (Figure 2).

1.1 For AHH-1 cells


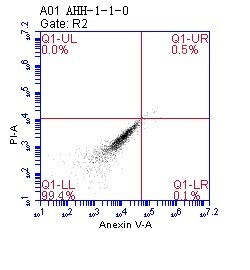

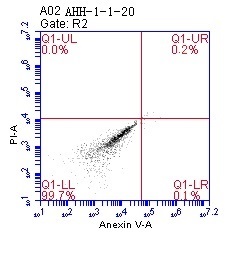

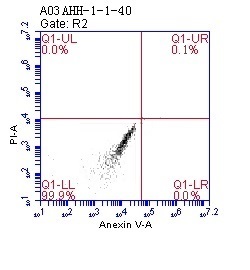

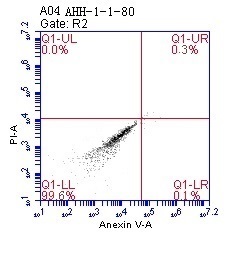

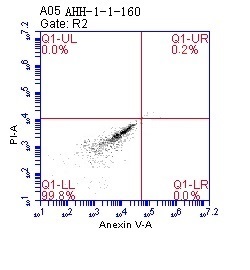


1.2 For HIEC cells


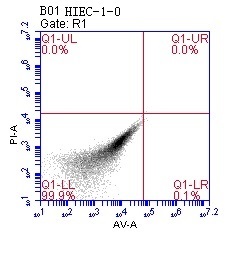

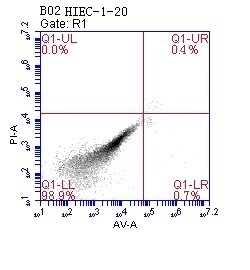

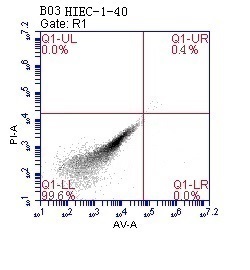

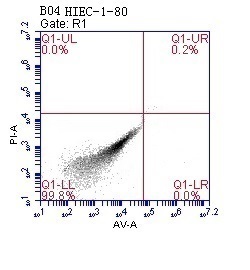

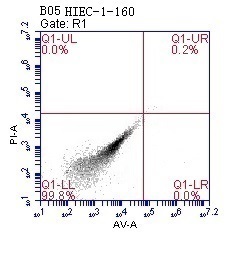


1. AHH-1 and HIEC cells were respectively administered to zymosan (tlrl-zyn, InvoGen) at 0, 20, 40, 80 and 160 μg/ml at 48h (Figure 2).

2.1 For AHH-1 cells


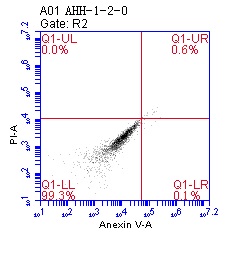

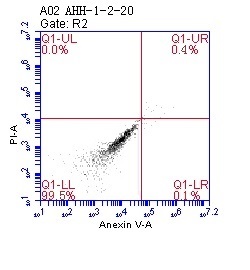

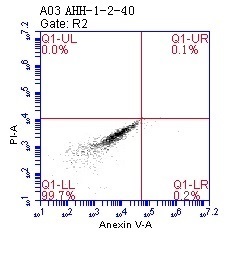

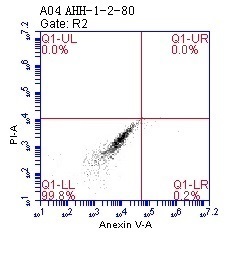

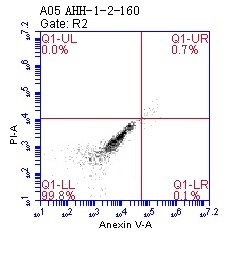


- 1. For HIEC cells


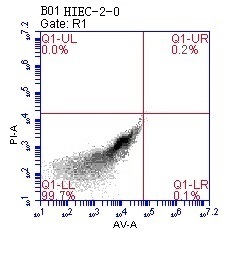

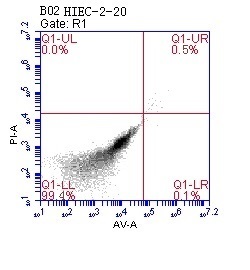

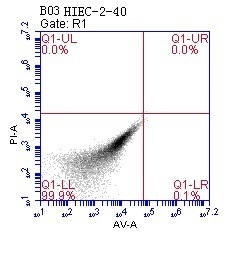

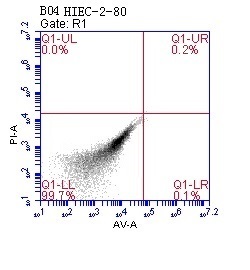

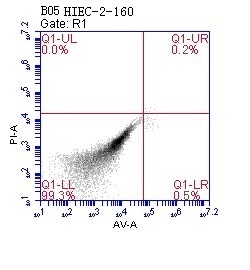


1. AHH-1 and HIEC cells were respectively administered to zymosan (tlrl-zyn, InvoGen) at 0, 20, 40, 80 and 160 μg/ml at 72h (Figure 2).

3.1 For AHH-1 cells


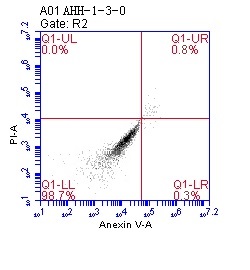

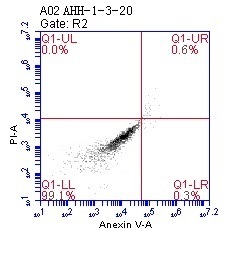

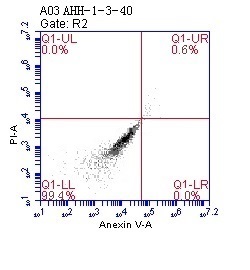

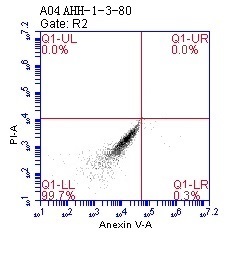

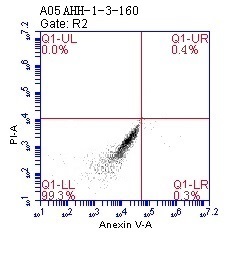


3.2 For HIEC cells


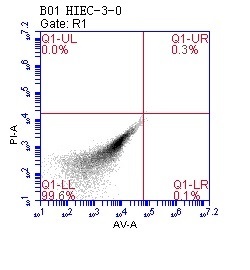

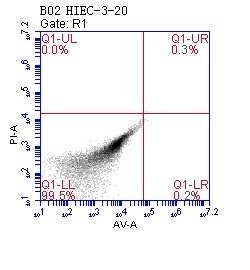

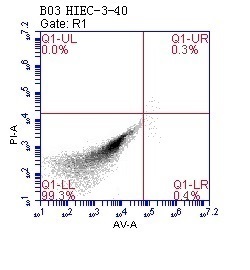

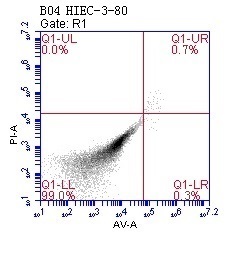

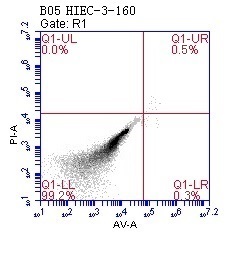

Supplement: Supplementary file 1 — Additional file 1. [file 40360_2021_482_MOESM1_ESM.docx]
